# Supplementary material for: “All of the things to everyone everywhere”: A mixed methods analysis of community perspectives on equitable access to monoclonal antibody treatment for COVID-19
Source: PLoS One. 2022 Nov 23;17(11):e0274043. doi: 10.1371/journal.pone.0274043 (PMC9683597; doi:10.1371/journal.pone.0274043)
Supplement: S1 Appendix — S1A File. Community Survey–English. S1B File. Community Survey–Spanish. (ZIP) [file pone.0274043.s001.zip › S1A Community Survey - English.docx]

**Community Survey on Tests and Treatment for COVID-19**

Please complete the survey below. Thank you!

Thank you for your interest in this study. Before completing the survey, it would be helpful to see if you are eligible to participate by asking you a few questions. These will ask you about your age and residence. The pre-screening questions should only take a few minutes to complete. If you are eligible, you will be taken directly to the full survey.

You do not have to answer any questions that you would not like to answer, but without answers to these questions you will not be eligible to participate in the study. No information about your identity, such as name, will be recorded until you are determined to be eligible for the study; at that time, this information will be kept secure. If you are not eligible for this study, we will keep the information collected during this pre-screening, but there will be no way for anyone to link that information to you.

If you have any questions or concerns, you can contact COMIRB, the Ethics Board that oversees our research, at 303-724-1055.

**Screening Questions**

1. What is your age in years?
2. Are you currently living in Colorado? Yes No
3. Based on your age or health status, are you someone Yes

who might be at higher risk from COVID-19? Maybe/Not sure No


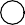

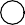

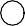

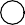

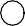


1. Are you a person who makes health care decisions Yes

for someone else who might be at higher risk from Maybe/Not Sure


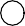

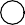

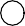


COVID-19, such as a spouse, parent, or grandparent? No

You are not eligible to complete the rest of this survey. Thank you for your time!

Click here to end the survey

**Community Survey on Monoclonal Antibody Drugs to Treat COVID-19**

You are being asked to be in this research study because you are an adult between the ages of 18 and 79 who lives in Colorado and who might be at a higher risk from COVID-19 or you are a friend or family member of someone who might be at higher risk from COVID-19. If you join the study, you will participate in a brief online survey that will ask for your thoughts and opinions about treatments for COVID-19.

You will receive $25 in the form of a gift card as reimbursement for your time. This survey should take 5-10 minutes to complete. This research is being paid for by the National Center for Advancing Translational Sciences (NCATS).

There are no medical risks to you. You may get bored with answering some of the questions. There may be risks the researchers have not thought of.

This study is not designed to benefit you directly. We hope to learn about how to help Coloradans get treatment for COVID-19. The data we collect will be used for this study but may also be important for future research. Your data may be used for future research or distributed to other researchers for future study without additional consent if information that identifies you is removed from the data.

Every effort will be made to protect your privacy and confidentiality by:

Keeping names of participants confidential Keeping identifying information in a secured location accessible only to the research team Only describing general characteristics of participants in research reports You have a choice about being in this study. You do not have to be in this study if you do not want to be. You do not have to answer any question you do not want to. By proceeding with this survey, you are consenting to participate in the study.

If you have questions, you can contact Dr. Bethany Kwan at [bethany.kwan@cuanschutz.edu](mailto:bethany.kwan@cuanschutz.edu) or Vanessa Owen, MA at [vanessa.owen@cuanschutz.edu.](mailto:vanessa.owen@cuanschutz.edu) You can call or email to ask questions at any time.

You may have questions about your rights as someone in this study. If you have questions, you can call COMIRB (the responsible Institutional Review Board) at (303) 724-1055. We will provide you a copy of this consent script upon request.

**Tests and Medications for COVID-19**

**The following questions are about your thoughts, opinions, and personal experiences with tests and medications for COVID-19. Please answer the following questions as accurately as possible. There are no right or wrong answers.**

1. How much have you heard about each of the following?

Medical tests (e.g., nasal swab) to determine if a person currently has COVID- 19?

Nothing at all Unsure A little A moderate

amount


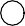

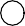

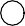

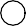


A lot


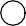


Vaccines to prevent a person
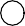

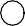

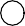

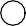

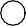
 from getting COVID-19?

Monoclonal antibody
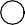

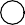

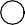

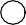

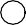
 medications to treat COVID-19?

Other medications to treat
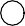

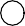

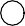

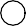

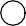
 COVID-19?

1b. What have you heard about monoclonal antibody medications to treat COVID-19?

1. Have you ever heard of the following monoclonal antibody medications (sometimes called "mAbs") for treating COVID-19?

Yes Unsure No

Bamlanivimab made by Eli Lilly
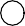

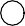

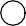


Bamlanivimab and etesevimab
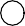

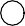

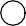
 made by Eli Lilly

Casirivimab and imdevimab
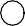

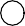

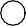
 made by Regeneron

Other "mAbs" or monoclonal
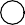

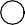

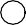
 antibody drugs for COVID-19

3a. Have you ever had COVID-19? Yes, I have had a positive test for COVID-19

Yes, I had symptoms after exposure to someone with confirmed COVID-19


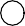

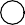


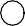
 Maybe, I suspect I have had COVID-19, but it was not confirmed


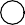
 No, not that I know of


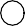

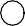


3b. Have you ever been hospitalized for COVID-19? Yes No

1. Have you ever received any of the following tests or medications for COVID-19?

Yes Unsure No

Medical tests (e.g., nasal swab)
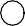

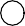

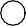
 to determine if you currently

have COVID-19?

Vaccines to prevent you from
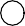

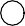

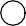
 getting COVID-19?

Monoclonal antibody
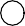

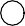

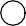
 medications to treat COVID-19?

Other drugs to treat COVID-19?
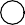

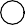

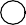


**Personal Preferences for COVID-19 Treatment**

1. If you were to get COVID-19, how worried are you Very worried


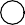

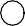

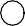

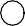


that you would end up in the hospital? Somewhat worried A little worried Not at all worried


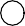

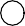

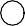


6a. If you were to get COVID-19 and a doctor Yes recommended you get a mAbs medication that may lessen Maybe the chance that you would be hospitalized, would you No agree to take that drug?

6b. Why would you not agree to take the mAbs medication?


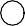

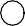

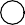


6c. mAbs medication is injected into your bloodstream Less willing through an IV tube for about one hour. How does that No difference affect your willingness to get the treatment? More willing

7. What question(s) would you have about mAbs treatment before deciding to get the treatment?

8a. At what types of infusion center locations would you be most comfortable receiving this treatment?

Yes Maybe No

Hospital
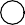

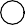

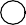
 Specialty Care Center
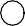

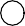

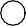
 Mobile Care Unit
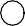

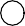

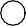
 Other place
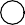

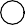

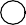


8b. At what other place(s) would you consider getting the mAbs medication?

9a. You previously indicated that you help make healthcare decisions for someone else who may be at high risk for COVID-19. Who is that person?

Parent Grandparent Adult child Spouse Other

9b. If other, please indicate their relationship to you here:

10. If that person were to get COVID-19, how worried Very worried

are you that they would end up in the hospital? Somewhat worried A little worried Not at all worried


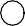

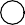

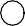

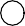

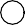

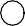

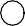


11a. If that person got COVID-19 and a doctor Yes recommended they get a mAbs medication that may lessen Maybe the chance that they would be hospitalized, would you No agree that they should take that medication?

11b. Why might you not agree?

1. What question(s) would you have before deciding if your friend/family member should get the mAbs treatment?


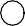

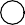

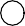


1. If a family member or friend were to get COVID-19, Yes who might be at higher risk from COVID-19, would you Maybe recommend they ask their doctor about how to get a No mAbs medication?
2. Based on what you currently know, how much do you trust that mAbs medications for COVID-19. (Check one for

each)

Trust a lot Trust some Trust a little Trust not at all Don't know

Are safe to take?
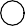

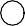

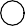

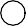

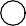


Will work to keep someone out
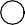

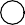

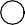

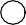

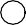
 of the hospital?

Won't make someone sick?

Will be available to those in your community who want it?

Will be affordable?

1. Do you have any additional comments or questions about mAbs medications for COVID-19?

**Personal Characteristics**

**Please tell us the following information to help us describe who completed our study.**

1. In what year were you born?

17a. What is your gender? Man

Woman Non-binary

I prefer to self-describe Prefer not to say

17b. If you prefer to self-describe please specify here:

18a. What is your race and ethnicity?

Black or African American White or Caucasian Hispanic or Latino/Latina Asian

Native Hawaiian or Pacific Islander Native American or Alaska Native Other (please specify)

(Select all that apply)

18b. If you selected other in the last question, please specify here:

19. What is the highest degree or level of education Some high school

you have completed? High school degree or GED Some college (no degree) 2 year college degree

4 year college degree Post graduate degree Prefer not to say

20a. What is your current employment status? Full-time Part-time

Contract/Temporary Unemployed and looking Unemployed and not looking Unable to work

Retired Other

I prefer not to say

20b. If you selected other on the last question, please specify here:

1. What is the ZIP code where you live?

**Current Health Status**

1. Have you ever been told by a doctor that you have...

Yes No Unsure

Diabetes Heart Disease Kidney Disease

Hypertension (High Blood Pressure)

Obesity (a body mass index 35 or higher)

Respiratory Disease (COPD, Asthma, etc.)

1. Are you currently taking medications that suppress Yes the immune system (e.g., chemotherapy, chronic No corticosteroids, anti-rejection medications, other

immunosuppressant medications)?

1. Do you currently have any health conditions Yes

associated with a compromised immune system (e.g., No HIV/AIDS, primary immunodeficiencies, splenectomy,

others)?

1. Overall, how would you rate your health at the Excellent

present time? Very good

Good Fair Poor

Would you be willing to be contacted for additional Yes, I am interested in being contacted about research study opportunities? For instance, we are future opportunities

looking for people who might be willing to do a longer No, I am not interested focus group on treatments for COVID-19. There might

also be additional survey opportunities. There would be additional compensation for other studies.

If you are willing to be contacted, please indicate that here:

If yes, please indicate the best way to reach you: Phone - call Phone - text Email

What is your preferred phone number?

What is your preferred email address?

Thank you for completing our survey about COVID-19. Once we have verified responses we will use the information below to send you a link to select and redeem your $25 e-gift card on a website called Tango Card. An e-gift card is electronic and will be delivered in an email; it can be printed and used in store, or used as a code that can be used online or shown in store. Remember to check your junk or spam folder in your email if you are having trouble finding the information on how to redeem your e-gift card.

Note, you may only complete the survey once. If responses appear to be duplicates or were not answered seriously, we may not send you a gift card.

What is your name?

(First and Last)

What email address should the e-gift card be sent to?

Please re-enter the email address your e-gift card should be sent to:

For verification purposes, please provide your mailing address:
