# Supplementary material for: “All of the things to everyone everywhere”: A mixed methods analysis of community perspectives on equitable access to monoclonal antibody treatment for COVID-19
Source: PLoS One. 2022 Nov 23;17(11):e0274043. doi: 10.1371/journal.pone.0274043 (PMC9683597; doi:10.1371/journal.pone.0274043)
Supplement: S2 Appendix — S2A File. Focus Group Guide–English. S2B File. Focus Group Guide–Spanish. (ZIP) [file pone.0274043.s002.zip › S2A Focus Group Guide - English.docx]

**Focus group Guide/English***

1. Today we’d like to focus on one particular type of COVID-19 treatment. What have you heard, if anything, about “monoclonal antibody treatments” for COVID-19? These are sometimes called “Monoclonal antibody” treatments or “Monoclonal antibody cocktails”, and go by names such as “bamlanivimab” or “Casirivimab” or “imdevimab” such as those made by the company Regeneron or Lilly”.

*I appreciate that is what you have heard. Thank you for sharing.* ***Show video.*** *Note that the purpose is not to critique the video but to give us a starting point for a conversation.*

- 1. Now that you’ve seen the video, what thoughts would you have about monoclonal antibodies?
  2. What do you think about Monoclonal antibody treatments? Is this something you might like to get for yourself? For a loved one? Why or why not?
  3. How do you think monoclonal antibody treatments might help other people in your community?
  4. What are your concerns about Monoclonal antibody treatment for COVID-19? (e.g., cost, burden, resources, risks of side effects, needing to delay vaccination)
  5. What do you know about how someone might get monoclonal antibody treatment in your community?

*Review logistics for how it works to get access in Colorado.* ***Share flyer.***

- 1. What might make it hard to get monoclonal antibody COVID-19 treatments in your community?
  2. If someone says they or a loved one has gotten monoclonal antibody treat ask them to share what went well/what could be improved about that process.

1. You have brought up a lot of questions and wonderings about monoclonal antibody treatments. Imagine you have just tested positive for Covid. What might you do next? How might you have a conversation with your doctor?
2. In your community, how might people find out about monoclonal antibody treatments for Covid-19?
   1. Where do community members get their health information?
   2. In general, how do you figure out if the information you get about new treatments for COVID-19 is trustworthy?
   3. How might we generate trust in your community about information about monoclonal antibody treatment?
   4. Who are the influencers in your community?
   5. Who do you trust to give you information about new treatments for COVID-19?
   6. What information do community members want and need to know about treatment for COVID-19? (e.g., cost, safety, insurance coverage)
   7. How should we get that information to them?

Given all the information needs you have mentioned about monoclonal antibody treatments what should we lead with/prioritize for education?

*That concludes our questions. But before we finish, we would like to know if there is anything we’ve missed. Is there anything else you think we should know?*

*A Spanish translation of this guide was use for the Spanish language focus group. The Spanish version is available on request
