# Supplementary material for: “All of the things to everyone everywhere”: A mixed methods analysis of community perspectives on equitable access to monoclonal antibody treatment for COVID-19
Source: PLoS One. 2022 Nov 23;17(11):e0274043. doi: 10.1371/journal.pone.0274043 (PMC9683597; doi:10.1371/journal.pone.0274043)
Supplement: S1 Appendix — S1A File. Community Survey–English. S1B File. Community Survey–Spanish. (ZIP) [file pone.0274043.s001.zip › S1B Community Survey - Spanish.doc]

*Página 1*

# Encuesta Comunitaria sobre las Pruebas y el Tratamiento de COVID-19

Por favor, rellene la siguiente encuesta.

El objetivo de este estudio de investigación es conocer la opinión de los habitantes de Colorado sobre los tratamientos de COVID-19. También queremos saber cómo ayudar a las personas a conseguir nuevos tratamientos para combatir el COVID-19.

¡Gracias!

Gracias por su interés en este estudio. Antes de completar la encuesta, sería útil determinar si usted cumple con los requisitos para participar, haciéndole algunas preguntas. En ellas se le preguntará por su edad y residencia. Las preguntas de preselección sólo deberían tardar unos minutos en completarse. Si cumple los requisitos, se le llevará directamente a la encuesta completa.

No tiene que responder a ninguna pregunta que no quiera contestar, pero si no responde a estas preguntas no podrá participar en el estudio. No se registrará ninguna información sobre su identidad, como por ejemplo su nombre, hasta que se determine que es elegible para el estudio; en ese momento, esta información se mantendrá segura. Si no cumple los requisitos para participar en este estudio, conservaremos la información recopilada durante esta preselección, pero no habrá forma de que nadie vincule esa información con usted.

Si tiene alguna pregunta o duda, puede ponerse en contacto con el COMIRB, el Comité de Ética que supervisa nuestra investigación, llamando al 303-724-1055.

## Preguntas de selección

1. ¿Cuál es su edad en años?

__________________________________

. ¿Vive actualmente en Colorado?

2

Sí

No

3

. Según su edad o estado de salud, ¿es usted alguien

Sí

que pudiera tener mayor riesgo de contraer COVID-19?

Quizás/No estoy seguro/a

No

4

. ¿Es usted una persona que toma decisiones sobre la atención

Sí

médica a nombre de alguien que podría estar en mayor riesgo de

Quizás/No estoy seguro/a

contraer COVID-19, como un cónyuge, padre/madre o abuelo/abuela?

No

No cumple con los requisitos para completar el resto

Haga clic aquí para finalizar la encuesta

de esta encuesta. ¡Gracias por su tiempo!

## Encuesta comunitaria sobre los medicamentos de anticuerpos monoclonales para tratar la enfermedad de COVID-19

Se le pide que participe en este estudio de investigación porque usted es una persona adulta de entre 18 y 79 años que vive en Colorado y que podría estar en mayor riesgo de contraer COVID-19 o es amigo/a o familiar de alguien que podría estar en mayor riesgo de contraer COVID-19. Si decide formar parte del estudio, participará en una breve encuesta en línea en la que se le pedirán sus ideas y opiniones sobre los tratamientos para COVID-19.

En compensación por su tiempo, recibirá $25 en forma de tarjeta de regalo. Esta encuesta debería durar entre 5 y 10 minutos. Esta investigación está financiada por el National Center for Advancing Translational Sciences (NCATS o Centro Nacional para el Avance de las Ciencias Translacionales).

No hay riesgos médicos para usted. Es posible que se aburra de responder a algunas de las preguntas. Puede haber riesgos en los que los investigadores no hayan pensado.

Este estudio no está diseñado para beneficiarle directamente. Esperamos aprender cómo ayudar a los habitantes de Colorado a recibir tratamiento para COVID-19. La información que recojamos se utilizará para este estudio, pero también puede ser importante para futuras investigaciones. Sus datos pueden ser utilizados para futuras investigaciones o distribuidos a otros investigadores para futuros estudios sin necesidad de un consentimiento adicional en caso de que se eliminen los datos que lo identifiquen.

Se hará todo lo posible para proteger su privacidad y confidencialidad:

Mantener la confidencialidad de los nombres de los participantes Mantener la información de identificación en un lugar seguro al que sólo pueda acceder el equipo de investigación Describir únicamente las características generales de los participantes en los informes de investigación Usted puede elegir si desea participar en este estudio. No tiene que participar en este estudio si no lo desea. No tiene que responder preguntas de este estudio si no lo desea. Al seguir adelante con esta encuesta, está dando su consentimiento para participar en el estudio.

Si tiene preguntas, puede ponerse en contacto con la Dra. Bethany Kwan escribiendo a bethany.kwan@cuanschutz.edu o con Vanessa Owen, MA escribiendo a vanessa.owen@cuanschutz.edu. Puede llamar o enviar un correo electrónico con sus preguntas en cualquier momento.

Es posible que tenga preguntas sobre sus derechos como participante en este estudio. Si tiene dudas, puede llamar al COMIRB (la Junta de Revisión Institucional responsable) al (303) 724-1055. Le proporcionaremos una copia de este guión de consentimiento si lo solicita.

## Pruebas y medicamentos para combatir COVID-19

**Las siguientes preguntas son sobre sus pensamientos, opiniones y experiencias personales con las pruebas y los medicamentos para COVID-19. Por favor, responda a las siguientes preguntas con la mayor precisión posible. No hay respuestas correctas o incorrectas.**

1. ¿En qué medida ha oído hablar de cada uno de los siguientes aspectos?

¿Pruebas médicas (por ejemplo, hisopo nasal) para determinar si una persona tiene actualmente COVID- 19?

Para nada

No estoy seguro/a

Muy poco

De manera

moderada

Mucho

¿Vacunas para evitar que una persona se contagie de COVID-19?

¿Medicamentos de anticuerpos monoclonales para tratar la COVID-19?

¿Otros medicamentos para tratar la COVID-19?

1b. ¿Qué ha oído sobre los medicamentos de anticuerpos

monoclonales para tratar la COVID-19?

__________________________________________

2. ¿Ha oído hablar de los siguientes medicamentos de anticuerpos monoclonales (a veces llamados "mAb") para tratar la enfermedad COVID-19?

Sí

No estoy seguro/a

No

Bamlanivimab producido por Eli Lilly

Bamlanivimab y etesevimab

producidos por Eli Lilly

Casirivimab y imdevimab

producidos por Regeneron

Otros fármacos "mAb" o anticuerpos monoclonales para combatir COVID-19

3

a. ¿Ha tenido alguna vez COVID-19?

Sí, he tenido una prueba positiva de COVID-19

Sí, tuve síntomas después de la exposición a alguien

con un caso confirmado de COVID-19

Tal vez, sospecho que he tenido COVID-19, pero

no fue confirmado

No que yo sepa

3

b. ¿Ha sido hospitalizado/a por COVID-19?

Sí

No

4. ¿Ha recibido alguna de las siguientes pruebas o medicamentos para COVID-19?

¿Pruebas médicas (por ejemplo, hisopo nasal) para determinar si usted tiene actualmente COVID- 19?

Sí

No estoy seguro/a

No

¿Vacunas para evitar que usted se contagie de COVID-19?

¿Medicamentos de anticuerpos monoclonales para tratar la enfermedad COVID-19?

¿Otros medicamentos para tratar COVID-19?

## Preferencias personales para el tratamiento con COVID-19

5

. Si usted se contagiara de COVID-19, ¿cuánto le preocupa

Me preocupa mucho

que pudiera ser internado/a en el hospital?

Me preocupa un poco

Me preocupa muy poco

No me preocupa para nada

6

a. Si usted recibiera COVID-19 y un médico

Sí

le recomendase una medicación mAb que podría disminuir

Quizás

la probabilidad de una hospitalización, ¿estaría

No

dispuesto/a a tomar ese fármaco?

6b. ¿Por qué no aceptaría tomar los medicamentos mAb?	__________________________________

6c. La medicación de los mAb se inyecta en el torrente sanguíneo	Menos dispuesto/a	a través de una sonda intravenosa durante aproximadamente una hora. No hay diferencia

¿Cómo afecta eso su disposición para recibir el tratamiento?	Más dispuesto/a

7. ¿Qué pregunta(s) tendría sobre el tratamiento

con mAb antes de decidir someterse a él?

__________________________________________

8a. ¿En qué tipo de centros de infusión se sentiría más cómodo/a para recibir este tratamiento?

Sí

Quizás

No

Hospital

Centro de atención especializada

Unidad móvil de atención médica

Otro lugar

8

b. ¿En qué otro(s) lugar(es) consideraría someterse

al tratamiento con medicamentos mAb?

__________________________________

9

a. Usted indicó previamente que ayuda con la toma de

Padre/Madre

decisiones de salud a otra persona que puede estar en

Abuelo/Abuela

alto riesgo de contraer COVID-19. ¿Quién es esa persona?

Hijo/a adulto/a

Cónyugue

Otra

9b. Si es otra persona, indique su relación con ella aquí:

__________________________________

10

. Si esa persona se contagiara de COVID-19, ¿en qué medida

Me preocupa mucho

le preocupa que pudiera ser internado/a en el hospital?

Me preocupa un poco

Me preocupa muy poco

No me preocupa para nada

11

a. Si esa persona tiene COVID-19 y un médico

Sí

le recomendase una medicación mAb que podría disminuir

Quizás

la probabilidad de una hospitalización, ¿estaría

No

de acuerdo en que debería tomar esa medicación?

11

b. ¿Por qué podría no estar de acuerdo?

__________________________________

12. ¿Qué pregunta(s) tendría antes de decidir si

su amigo/a o familiar debería recibir el tratamiento con mAb?

__________________________________________

13

. Si un miembro de la familia o amigo/a se contagia

Sí

de COVID-19, y esa persona pudiera tener mayor riesgo de

Quizás

contraer COVID-19, recomendaría que preguntaran a su médico sobre cómo obtener medicamentos mAb?

No

14. Basándose en lo que sabe actualmente, ¿hasta qué punto confía en que los medicamentos mAb para COVID-19....? (Marque una respuesta para cada pregunta)

Confío mucho

Confío en cierta medida

Confío un poquito

No confío para nada

No sé

Son seguros de tomar

Ayudarán a alguien a

salir del hospital

No harán enfermar a nadie

Estarán disponibles en su comunidad

para aquellas personas que los quieran

Serán asequibles

15

. ¿Tiene algún comentario o pregunta adicional

sobre los medicamentos mAb para combatir la COVID-19?

__________________________________________

## Características personales

**Por favor, facilite la siguiente información para ayudarnos a describir quién ha completado nuestro estudio.**

16. ¿En qué año nació?

__________________________________

17

a. ¿Cuál es su sexo?

Hombre

Mujer

No binario

Prefiero autodescribirme

Prefiero no decirlo

17b. Si prefiere autodescribirse, por favor, especifique

aquí:	__________________________________

18

a. ¿Cuál es su raza y etnia?

Negro/a o Afroamericano/a

Blanco/a o caucásico/a

Hispano/a o Latino/a

Asiático/a

Nativo/a de Hawái/Islas del Pacífico

Nativo/a americano/a o de Alaska

Otra (por favor, especifique)

(Seleccione todas las que correspondan)

18

b. Si seleccionó "otra" en la última pregunta,

por favor especifique aquí:

__________________________________

19

. ¿Cuál es el grado o nivel de educación más alto

Algo de secundaria

que ha completado?

Título de secundaria o GED

Algunos estudios universitarios (sin título)

título

universitario de 2 años

universitario de 4 años

título

Título de posgrado

Prefiero no decirlo

20

a. ¿Cuál es su situación laboral actual?

A tiempo completo

A tiempo parcial

Por contrato/temporal

Desempleado/a y buscando trabajo

Desempleado/a y no buscando trabajo

Incapacitación laboral

Retiro

Otra

Prefiero no decirlo

20

b. Si seleccionó "otra" en la última pregunta,

por favor especifique aquí:

__________________________________

21. ¿Cuál es el código postal de la zona donde vive?

__________________________________

## Estado de salud actual

. ¿Alguna vez le ha dicho un médico que tiene...?

22

Sí

No

No estoy seguro/a

Diabetes

Enfermedad del corazón

Enfermedad renal

Hipertensión (Presión arterial

alta)

Obseidad (un índice de masa

corporal de 35 o superior)

Enfermedad respiratoria (COPD, asma, etc.)

23. ¿Está tomando actualmente medicamentos que suprimen	Sí

el sistema inmunológico (por ejemplo, la quimioterapia, los	No

corticosteroides, medicamentos compensatorios, otros medicamentos inmunosupresores)?

24. ¿Tiene actualmente algún problema de salud asociado con	Sí

un sistema inmunológico comprometido (por ej., VIH/SIDA,	No

inmunodeficiencias primarias, esplenectomía, otros)

25

. En general, ¿cómo calificaría su salud

Excelente

en la actualidad?

Muy buena

Buena

Regular

Mala

Sí, estoy interesado/a en que se pongan en contacto

conmigo respecto a oportunidades futuras

No, no estoy interesado/a

¿Estaría dispuesto/a a que nos pusiéramos en contacto con usted para otras oportunidades de estudios investigativos? Por ejemplo, estamos buscando personas para participar en grupos de enfoque más extensos acerca de tratamientos para COVID-19. Podrían haber otras oportunidades de encuestas. Habría una compensación adicional por los otros estudios. Si está dispuesto/a a ser contactado/a, indíquelo aquí:

En caso afirmativo, indique la mejor forma de contactar con usted:

Teléfono - llamada

Teléfono - mensaje de texto

Email

¿En qué número de teléfono prefiere que le contactemos?

__________________________________

¿A qué dirección de correo electrónico prefiere que le escribamos?

__________________________________

Gracias por completar nuestra encuesta sobre COVID-19. Una vez que hayamos verificado las respuestas, utilizaremos la información que figura a continuación para enviarle un enlace para seleccionar y canjear su tarjeta electrónica de regalo de $25 en un sitio web llamado Tango Card. Una tarjeta electrónica de regalo se enviará por un correo electrónico; puede imprimirse y utilizarse en tiendas, o utilizarse como un código que puede usarse compras en línea o mostrarse en tiendas. Recuerde revisar la carpeta de correo no deseado o “spam” de su email si tiene problemas para encontrar la información sobre cómo canjear su tarjeta electrónica de regalo.

Tenga en cuenta que sólo puede completar la encuesta una vez. Si las respuestas parecen ser duplicadas o no fueron contestadas con seriedad, podríamos no enviarle una tarjeta de regalo.

| ¿Cuál es su nombre? | __________________________________  (Nombre y apellido) |
| --- | --- |
| ¿A qué dirección de correo electrónico debe enviarse la tarjeta electrónica de regalo? | __________________________________ |
| Vuelva a introducir la dirección de correo electrónico a la que debe enviarse su tarjeta electrónica de regalo: | __________________________________ |

A efectos de verificación, facilite su dirección

postal:	__________________________________
