# Supplementary material for: “All of the things to everyone everywhere”: A mixed methods analysis of community perspectives on equitable access to monoclonal antibody treatment for COVID-19
Source: PLoS One. 2022 Nov 23;17(11):e0274043. doi: 10.1371/journal.pone.0274043 (PMC9683597; doi:10.1371/journal.pone.0274043)
Supplement: S2 Appendix — S2A File. Focus Group Guide–English. S2B File. Focus Group Guide–Spanish. (ZIP) [file pone.0274043.s002.zip › S2B Focus Group Guide - Spanish.doc]

**Guía de facilitación de grupos de enfoque**

Bienvenida

Hola a todos. Gracias por formar parte de nuestro grupo de discusión hoy.

Presentaciones

Mi nombre es _____ y mis pronombres son ___. Este/a es (nombre del/la cofacilitador/a).

Básicamente, estamos aquí hoy para aprender de ustedes. Nuestro objetivo es mejorar el acceso a un tratamiento específico para la enfermedad de COVID-19. Vamos a

- revisar algunas reglas básicas
- darles la oportunidad de presentarse
- contarles un poquito más de nuestro proyecto
- contarles un poquito más del tratamiento nMAb
- responder cualquier pregunta que tengan
- hacerle algunas preguntas para ayudarnos a entender lo que ustedes tienen en cuenta a la hora de tomar decisiones sobre la atención médica
- contestar cualquier pregunta restante
- darles las instrucciones para solicitar su tarjeta de regalo

Nuevamente, mi nombre es _______. Mi papel en este proyecto es _______________

Reglas básicas

Les voy a contar un poco lo que suele ocurrir en un grupo de discusión como este.

1) Mi papel es hacer preguntas y estimular la participación. Serán ustedes quienes hablen más, pero puede que les pida que se expliquen más a fondo o que den un ejemplo. Haré todo lo posible para que todos tengan la oportunidad de compartir y también para que sigamos avanzando y podamos obtener respuestas detalladas a todas las preguntas. Tenemos hasta la(s) (decir hora aquí), lo que significa que quizá tenga que interrumpir a alguien para que haya tiempo para que otros den su opinión. Así que esa es la primera regla básica: tómese el tiempo necesario para dar una respuesta meditada y deje tiempo para que los demás hagan lo mismo.

2) En segundo lugar, ¡NO HAY RESPUESTAS CORRECTAS O INCORRECTAS! Todos ustedes tienen experiencias y opiniones, y por eso estamos aquí. Queremos conocer una amplia gama de opiniones sobre las preguntas que formularemos. Así que, por favor, díganos lo que piensa, aunque sea para discrepar.
3) A veces surgen temas delicados en estas sesiones, por lo que queremos estar seguros de que todos lo entienden y aceptan la confidencialidad. NADA de lo que se comunique en esta sala se repetirá a nadie que no haya formado parte de esta conversación. ¿Y si vuelve a casa y alguien le pregunta qué tal fue? Dígales que ha acordado mantener la confidencialidad. Les pido a cada uno/a de ustedes que digan SÍ para indicar que están de acuerdo con la regla de confidencialidad. Si por alguna razón no pueden dar un SÍ, por favor, sepan que estamos agradecidos por su tiempo, pero entiendan que tendremos que pedirles que abandonen el grupo.
4) Hasta ahora, hemos hablado de la importancia de asegurarse de que todo el mundo tiene tiempo para responder a cada pregunta, de que no hay respuestas correctas o incorrectas y de que la confidencialidad es necesaria. Ahora toca hablar de la grabación. Tenemos una grabadora de audio y grabamos los grupos de discusión porque queremos estar seguros de que captamos todo lo que dicen. Y ninguno/a de nosotros/as puede escribir tan rápido. Las únicas personas que tendrán acceso a la grabación son el equipo de investigación y el/la transcriptor/a, que hará una versión escrita detallada de todo lo que se dijo. No utilizaremos ningún nombre ni detalle identificativo.

Como estamos haciendo una grabación de audio, es MUY IMPORTANTE que hablemos UNO POR UNO/UNA POR UNA, para que tengamos una grabación clara. Cuando empecemos a grabar, le pediré a cada uno/a que dé un SÍ verbal si está de acuerdo en ser grabado/a.

5) Lo último que le pedimos como norma básica es que por favor apague su teléfono móvil o lo ponga en modo silencioso.

Tenemos previsto terminar a la(s) (hora), pero antes de empezar a grabar, ¿hay alguna pregunta sobre las normas básicas?

Establecer relaciones

Ahora que estamos grabando, por favor dé un SÍ verbal para que sepamos que acepta ser grabado/a.

Gracias.

Entremos en las presentaciones. Diga su nombre, comparta sus pronombres sólo si lo desea, y cuéntenos de una comida excelente que recuerde. Comenzaremos nosotros/as.

El objetivo de nuestro proyecto es hacer todo lo posible para que las personas de sus comunidades tengan acceso a tratamientos eficaces contra COVID-19 y al tratamiento con nMAb en particular.

Bien, ahora pasaremos a las preguntas específicas sobre sus percepciones sobre el proceso de nMAb.

Tenemos un folleto que da una visión general, pero primero:

**Guía para preguntas**

**Atributos de la innovación**

*Definición: Cómo ve el adoptante potencial los pros y los contras de la innovación*

1. ¿Qué sabe sobre los tratamientos disponibles de COVID-19?
   a. ¿Cómo obtuvo esa información?
2. Hoy nos centraremos en un tipo particular de tratamiento de COVID-19. ¿Qué ha oído, si es que ha oído algo, sobre los "tratamientos con anticuerpos monoclonales" para la enfermedad COVID-19? FOLLETO AQUÍ.
3. A veces, estos tratamientos se denominan tratamientos "MAb" o "cócteles MAb"
   a. La Administración de Alimentos y Fármacos de EE.UU. ha emitido lo que se denomina una "autorización de uso de emergencia" para el tratamiento con MAb para personas con una enfermerdad COVID-19 de leve a moderada. ¿Qué opina al respecto?
   b. ¿Qué le parecen los tratamientos con MAb? ¿Es algo que le gustaría conseguir para usted? ¿Para un ser querido?
   c. ¿Cómo podría beneficiar el tratamiento con MAb a las personas de su comunidad?
   d. ¿Cuáles son sus preocupaciones sobre el tratamiento con MAb para la COVID-19? (por ejemplo, el costo, la dificultad, los recursos, los riesgos de los efectos secundarios, la necesidad de retrasar la vacunación) ¿Cuáles podrían ser las preocupaciones de otras personas en su comunidad?
   e. ¿Cómo cree que alguien podría acceder al tratamiento con MAb en Colorado?
   f. ¿Ha oído hablar del sistema de registro y asignación aleatoria?
   g. ¿Qué preguntas tiene sobre el tratamiento con MAb para la COVID-19?

**Adoptantes previstos**

*Definición: Aquellos destinados a adoptar y promulgar innovaciones*

4. ¿Qué motivaría a la gente de su comunidad a participar en el tratamiento con MAb para combatir COVID-19?
   a. Piense en el lugar donde vive. ¿Cuáles son algunas de las formas en las que la ubicación podría ser un obstáculo para recibir tratamiento para COVID-19?
   b. ¿Existen factores culturales en su comunidad que hagan que la gente sea reacia a recibir tratamiento de COVID-19?
   c. ¿Qué otras cosas podrían dificultar el acceso de los miembros de su comunidad a los tratamientos de COVID-19?
   d. ¿Puede hablarnos un poco del enfoque de su comunidad sobre la atención sanitaria? ¿Existen valores u objetivos específicos relacionados con la salud?
   e. ¿Cuáles son algunas de las formas en que las personas de su comunidad establecen contacto entre sí?
   f. ¿Cómo se informa la gente de su comunidad sobre la atención médica?
   g. ¿Confía su comunidad en el sistema de salud? ¿Sabe por qué/por qué no?
   h. Cuando se trata de información sobre la salud, ¿en quién confían los miembros de la comunidad?
   i. Cuando piensa en su comunidad, ¿quiénes son las personas más propensas a participar en el tratamiento de COVID-19? ¿Quiénes tendrían menos posibilidades de participar?
   j. ¿Qué haría falta para que la gente de su comunidad se sintiera segura al inscribirse en el tratamiento de COVID-19?

¿Cómo se sienten? ¿Necesitan tomar un descanso? Hagamos una pausa de cinco minutos.

**Comunicación e influencia**

*Definición: El uso de canales de marketing y comunicación para orientar una difusión formal y planificada, dirigida a los adoptantes previstos*

5. Cuando piensa en hacer llegar la información sobre el tratamiento de la COVID-19 a la gente de su comunidad, ¿qué cree que podría funcionar?
   a. ¿Cómo decide si puede confiar en la información sobre los nuevos tratamientos para COVID-19?
   b. ¿En quién confía para que le dé información sobre los nuevos tratamientos para COVID-19?
   c. ¿Existen personas, sistemas u organizaciones que hayan generado confianza en su comunidad? ¿Quién tiene la mayor influencia? Cuando tienen algo importante que compartir, ¿cómo llegan a la comunidad?
   d. ¿Hay personas en su comunidad que apoyen el proceso de nMAb? ¿Quiénes son? ¿Qué es lo que dicen?
   e. ¿Hay personas en su comunidad que estén en contra del proceso de nMAb? ¿Quiénes son? ¿Qué es lo que dicen?
   f. ¿Qué información sería importante para su comunidad sobre los tratamientos para COVID-19? (por ejemplo, costo, seguridad, cobertura del seguro)
   g. ¿Qué ha oído decir a otras personas de su comunidad sobre el tratamiento con MAb para COVID-19?
   h. ¿Qué opinan los líderes de su comunidad sobre el tratamiento con MAb para COVID-19?
   i. ¿Conocen los médicos de su comunidad el tratamiento con MAb para COVID-19? ¿Qué han dicho los médicos u otros profesionales de la medicina sobre los tratamientos con MAb? ¿Recomiendan este tratamiento?

**Proceso de ejecución (puesta en marcha)**

*Definición: El proceso por el que una nueva innovación se difunde en una organización*

6. ¿Qué determina que se utilice el proceso de nMAb en su comunidad?
   a. ¿Cuáles son algunas formas de hacer llegar la información sobre los nMAb a la gente de su comunidad?
   b. En su comunidad, ¿hay tensión acerca de cómo acceder al tratamiento con nMAb? ¿Qué puede decirnos al respecto?
   c. ¿Quién debería participar en los debates sobre la organización del proceso de nMAb en su comunidad?
   d. Piense en el sistema de prestación de asistencia sanitaria de su comunidad. ¿Podría ese sistema ayudar a superar las barreras al tratamiento con nMAb?
   e. Siguiendo con la idea del sistema sanitario, ¿cómo podrían favorecer el tratamiento con nMAb?
   f. ¿Cuáles son algunas de las formas en que su comunidad puede proporcionar comentarios e información sobre el proceso de nMAb?
   g. ¿Cuáles son algunas de las formas en que los miembros de la comunidad pueden obtener información sobre el progreso del proceso de nMAb?

Esas son todas las preguntas que tenemos. ¿Nos hemos olvidado de algo? ¿Qué otra cosa creen que deberíamos saber?

Gracias por formar parte de este grupo de discusión. Un par de cosas antes de culminar:

Tenemos una breve encuesta de salida. Contiene algunas preguntas sobre la demografía y sus perspectivas generales sobre la participación en este grupo.

También estamos invitando a los participantes a otro grupo de discusión que trata específicamente de cómo la gente de su comunidad encuentra información. Los participantes nos ayudarán a tomar algunas decisiones sobre el tipo de medios de comunicación en los que invertir y otras formas de hacer llegar la información a su comunidad. Habrá una compensación adicional por participar en ese grupo. Si les interesa, hay espacio en la encuesta de salida para informárnoslo.

Y finalmente, aquí tienen cómo conseguir sus tarjetas de regalo.

**Referencias**

Solórzano, D. G., & Yosso, T. J. (2002). Critical race methodology: Counter-storytelling as an analytical framework for education research. (Metodología crítica de la raza: La contrahistoria como marco analítico para la investigación educativa.) *Qualitative inquiry*, *8*(1), 23-44.

Tuck, E., & Yang, K. W. (2014). R-words: Refusing research. (Palabras R: Rechazando la investigación)  En *Humanizing research: Decolonizing qualitative inquiry with youth and communities* (Descolonizar la investigación cualitativa con los jóvenes y las comunidades), p. 223-248.
